# Clinical characteristics and prognostic analysis of postoperative recurrence or metastasis of low-risk gastrointestinal stromal tumors

**DOI:** 10.1186/s12957-024-03339-z

**Published:** 2024-02-23

**Authors:** Lianlian Cao, Chen Lin, Yu Liu, Chao Sui, Zhaoping Li, Li Chen, Wenxian Guan, Liang Tao, Tingting Tao, Meng Wang, Feng Wang

**Affiliations:** 1 Division of Gastric Surgery, Department of General Surgery, Nanjing Drum Tower Hospital, Drum Tower Clinical Medical College, Nanjing University of Chinese Medicine, Nanjing, China; 2 Division of Gastric Surgery, Department of General Surgery, Nanjing Drum Tower Hospital, Affiliated Hospital of Medical School, Nanjing University, Nanjing, China; 3 Department of General Surgery, Nanjing Drum Tower Hospital, Affiliated Hospital of Medical School, Nanjing University, Nanjing, China

**Keywords:** Gastrointestinal stromal tumor, Low-risk, Recurrence, Metastasis

## Abstract

**Background:**

Gastrointestinal stromal tumors (GISTs) are the most common mesenchymal tumors of the digestive tract. This study aimed to investigate the clinical characteristics and prognosis of postoperative recurrence or metastasis in patients with low-risk stromal tumors, in order to take individualized postoperative management and treatment for patients with low-risk GISTs with relatively high recurrence.

**Methods:**

We retrospectively analyzed the clinicopathological and follow-up data of patients with GISTs who underwent surgical resection in Nanjing Drum Tower Hospital from March 2010 to December 2021. A total of 282 patients with low-risk GISTs were included, none of whom were treated with imatinib. Univariate and multivariate Cox analysis and survival curves were used to explore the relationship between clinical features and recurrence or metastasis in patients with low-risk GISTs.

**Results:**

Of the 282 patients with low-risk GISTs who met inclusion criteria, 14 (4.96%) had recurrence or metastasis. There was a correlation between tumor size, primary site, resection type, Ki67 index, neutrophil lymphocyte ratio (NLR) and CD34 expression and postoperative recurrence or metastasis of GISTs (*P* < 0.05). Subsequently, multifactorial analysis showed that tumor primary site, tumor size, and Ki67 index were independent risk factors affecting postoperative recurrent or metastasis in patients with low-risk GISTs (*P* < 0.05). Ultimately, According to Kaplan-Meier analysis, non-gastric primary tumors, larger tumors, and high Ki67 index were significantly associated with poor progression-free survival ( PFS ).

**Conclusions:**

Tumor location, tumor size and Ki-67 were independent risk factors for postoperative recurrence and metastasis in patients with low-risk GISTs. Based on the 2008 modified NIH recurrence risk grading system, combined with the above three factors, it can be used to evaluate the prognosis of patients with low-risk GISTs and provide personalized postoperative review and follow-up management recommendations.

## Introduction

Gastrointestinal stromal tumor ( GIST ) is a rare tumor derived from interstitial cells of Cajal in the gastrointestinal tract. It is mostly caused by oncogenic activation mutations of KIT or PDGFRA genes [1]. The global incidence of GIST is estimated to be approximately 10-20 parts per million per year [2], and the disease predominantly affects the elderly population, with the median age of patients is roughly between 60 and 65 years old [3]. GIST can occur in any part of the gastrointestinal tract, especially in the stomach ( 60-65 % ) and small intestine ( 20-25 % ), and occasionally in other parts of the abdominal cavity [4]. At present, surgical resection is still the main treatment for primary GIST. Unfortunately, even after complete surgical removal of both localized and primary GISTs, a large number of patients still experience a recurrence within the first five years after treatment [5, 6]. Currently, the malignancy of tumors is considered a crucial risk factor for predicting recurrence and metastasis in GISTs [7]. Therefore, thorough exploration of additional prognostic factors related to recurrence risk stratification has the potential to ameliorate the precise assessment of patient prognosis.

According to the National Institutes of Health (NIH) in the United States' latest revised recurrence risk stratification system [8], GISTs originating from any site and with tumor sizes ranging from 2 to 5 cm and a mitotic index of ≤5 per 50 high-power fields (HPF) are classified as low-risk GISTs. Nevertheless, the malignancy potential cannot be entirely dismissed even in instances of smaller tumors or lower mitotic activity. In a study focusing on follow-up procedures for low-risk GISTs, it was revealed that approximately 5.7% of the patients experienced disease recurrence [9]. It is worth noting that national and international guidelines for the diagnosis and treatment of gastrointestinal stromal tumors recommend that patients with GISTs who have a moderate to high risk of recurrence should take oral imatinib as adjuvant therapy after surgery [10, 11]. These intermediate- and high-risk patients may be able to delay relapse with postoperative adjuvant imatinib [12], whereas patients with low-risk GISTs are not recommended for this regimen. However, clinical observations have found that even patients with low-risk GISTs often show a relatively good prognosis, there are still a few patients with recurrence or metastasis. Recent clinical studies have preliminarily noted that gastric stromal tumors with a tumor diameter of 2 to 5 cm are classified as low risk, but exogenous tumors have a worse prognosis than endogenous tumors and may be more likely to recur [13]. Nevertheless, current research tends to focus on the prognostic impact on patients with intermediate and high-risk GISTs, while research on patients with low-risk GISTs is relatively limited. Therefore, the main objective of this study was to analyze the clinical characteristics and prognosis of recurrence or metastasis in patients with low-risk GISTs who underwent radical resection.

## Materials and methods

### Patient section

This study retrospectively analyzed 282 patients with low-risk GIST who were diagnosed and treated at the Department of Gastrointestinal Surgery, Nanjing Drum Tower Hospital from 1 March 2010 to 31 December 2021, with complete clinical and follow-up data. A total of 268 patients who underwent primary stromal tumor resection and had no recurrence or distant metastasis during postoperative follow-up ( follow-up to February 2022 ) were included. In addition, 14 patients who had recurrence and metastasis during follow-up or received secondary surgery for recurrence and metastasis were also included. The diagnosis of GIST depends on Chinese and NCCN guidelines. The inclusion criteria were as follows: Aged 18-80 years old; surgical resection; diagnosis confirmed by postoperative pathology, immunohistochemistry and genotyping; classified as low-risk GISTs based on the modified NIH recurrence risk grading system; neither preoperative nor postoperative received any targeted therapy including imatinib; patients and close relatives informed consent. The excluded criteria were as follows: Local or systemic metastasis at the time of initial diagnosis; with other malignant tumors; clinical and follow-up data were incomplete.

### Study design

This is a single-center retrospective study. Its primary focus is on the characteristics of postoperative recurrence and metastasis in patients with low-risk stromal tumors and their prognosis. We used Progression-Free Survival (PFS) as the primary study endpoint, which was defined as the time from the date of initial surgery to the date of GIST progression or death. Furthermore, if there was no progression or death, the date of last follow-up was considered as the study outcome for PFS. Clinical data and histopathological parameters were obtained from medical records. We used a combination of outpatient records and telephone follow-up to conduct postoperative follow-up studies. The postoperative recurrence and metastasis of the patients were determined by outpatient review, and the metastasis or recurrence of the disease was confirmed by imaging examination. Our follow-up includes the patient's adjuvant treatment, recurrence and metastasis, survival and postoperative review, recording the time of recurrence, the organ metastasized, etc., and the time of death of the deceased patient.

### Statistical analysis

All relevant statistical analyses were performed using SPSS 22.0 software ( IBM Corporation, Armonk, NY, USA ). Continuous variables were compared using the independent samples t-test, and categorical variables were compared using the χ2 test or Fisher's exact test. The test efficiency and critical value of continuous variables such as tumor size, NLR and PLR were determined by receiver operating characteristic ( ROC ) curve analysis. Kaplan-Meier analysis was used to draw the survival curve of the two groups, and the difference in survival rate between subgroups was analyzed by log-rank test. Univariable and multivariable Cox regression was used to analyze factors associated with tumor recurrence or metastatic disease, and the 95% confidence interval of the HR (hazard ratio) was reported. *P* value < 0.05 was considered statistically significant.

## Results

### Patient characteristics

**Fig. 1:**
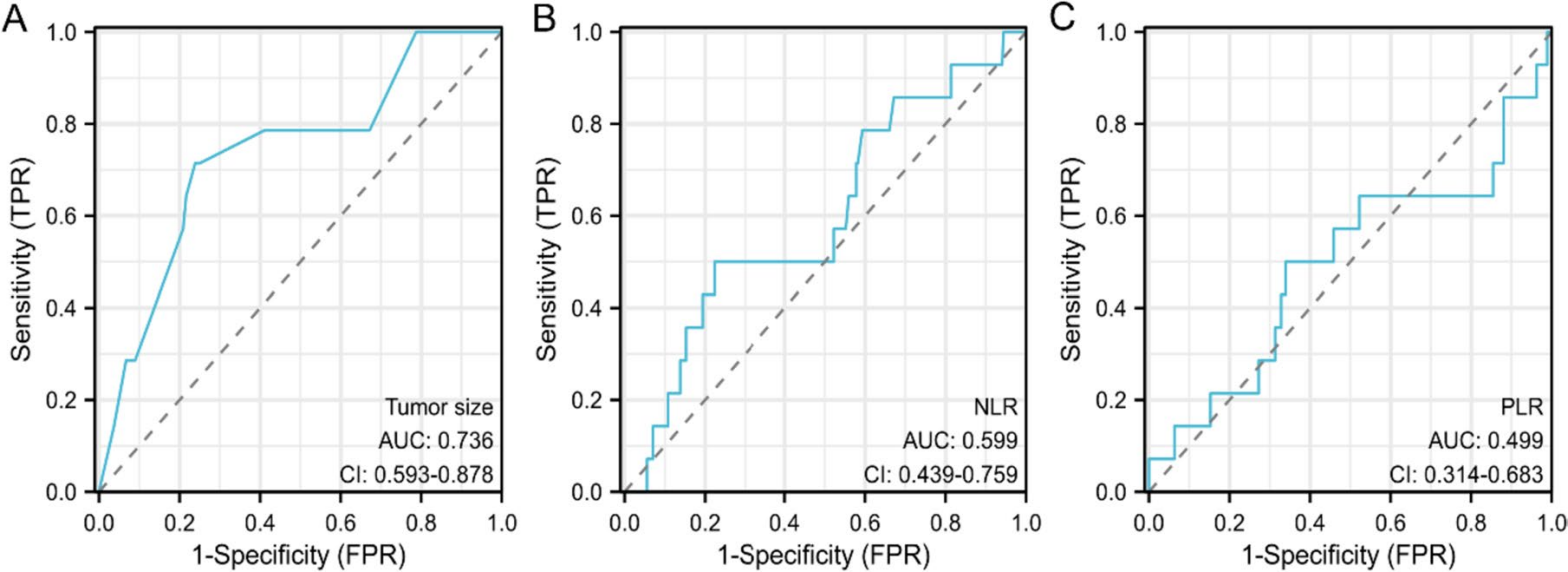
Receiver operator characteristic curve analysis of Tumor size, NLR, PLR. A Tumor size; B NLR; C PLR

Between 1 March 2010 and 31 December 2021, 282 patients were diagnosed with low-risk gastrointestinal stromal tumors and underwent therapeutic resection. Based on our previous clinical studies, preoperative inflammatory factors play a crucial role in determining the prognosis of intermediate and high-risk GIST patients. High levels of neutrophil-to-lymphocyte ratio (NLR) and platelet-to-lymphocyte ratio (PLR) have a negative correlation with relapse-free survival [14]. Consequently, we examined the effect of NLR and PLR as indicators of low-risk GISTs. Blood samples at the time of admission for the first diagnosis were used, which included indices such as platelet, lymphocyte and neutrophil counts to reflect the initial inflammatory state of the patients. Both NLR and PLR were calculated by blood routine and blood biochemical results before the first treatment. We plotted the ROC curve for tumor size (Fig. 1A). The area under the curve ( AUC ) of tumor size was 0.736, and the cut-off value was 4.0. Based on a similar method, we obtained an AUC of 0.599 and a cut-off value of 2.81 for NLR (Fig. 1B) and an AUC of 0.499 and a cut-off value of 153.31 for PLR (Fig. 1C). According to the cut-off values of these three indicators, they were divided into lower value group and higher value group for subsequent analysis.

Two hundred eighty-two patients with low-risk GIST participated in the study, of whom 14 (4.96%) were diagnosed with postoperative recurrence or metastasis. Among 14 recurrent patients, 6 cases (21%) experienced local recurrence (stomach, *n*=2; small intestine, *n*=2; duodenum, *n*=1, and rectum, *n*=1), while 8 cases (74%) exhibited distant metastasis. Specifically, 4 cases (50.0%) involved the liver, 2 cases (25.0%) involved the peritoneum, 1 case (12.5%) involved both the liver and peritoneum, and 1 case (12.5%) involved other sites. Additionally, 5 patients underwent a second surgical intervention after recurrent metastasis, while 5 patients initiated imatinib treatment upon confirmed recurrence. 3 patients were regularly reviewed without medication, and tragically, 1 patient died from the condition. Table 1 summarizes the comparison of clinicopathological features between patients with and without recurrence and metastasis, showing significant differences in tumor location (*P* < 0.001), resection type (*P* = 0.013), tumor size (*P* = 0.010), NLR (*P* = 0.018), CD34 (*P* = 0.002) and Ki67 index (*P* < 0.001). The primary sites of the tumor include stomach, small intestine, duodenum and other parts. We found that recurrence or metastasis was more common in male patients (64.3%) compared to female patients (35.7%). Furthermore, among low-risk GIST patients with recurrent metastatic disease, tumor size greater than 4 cm was the most common, representing 78.6% of cases.

**Table 1:** Characteristics of low-risk GIST patients with and without recurrence or metastasis

Characteristics	Patients without recurrence or metastasis group (*n*=268)	Patients with recurrence or metastasis group (*n*=14)	*P* value
**Age (%)**			0.595
≤ 60	151 (56.3)	9 (64.3)	
> 60	117 (43.7)	5 (35.7)	
**Gender (%)**			0.183
Male	122 (45.5)	9 (64.3)	
Female	146 (54.5)	5 (35.7)	
**Tumor site (%)**			**< 0.001**
Stomach	187 (69.8)	4 (28.6)	
Small intestine	59 (22.0)	6 (42.9)	
Duodenum	18 (6.7)	1 (7.1)	
Other	4 (1.5)	3 (21.4)	
**Resection type(%)**			**0.013**
Laparoscopic resection	131 (48.9)	2 (14.3)	
Open resection	137 (51.1)	12 (85.7)	
**Tumor size (%)**			**0.010**
<4 cm	158 (59.0)	3 (21.4)	
≥4 cm	110 (41.0)	11 (78.6)	
**CD117 (%)**			0.517
(-)	6 (2.2)	1 (7.1)	
(+)	58 (21.6)	4 (28.6)	
(++)	69 (25.7)	2 (14.3)	
(+++)	135 (50.4)	7 (50.0)	
**CD34 (%)**			**0.002**
(-)	13 (4.9)	4 (28.6)	
(+)	70 (26.1)	2 (14.3)	
(++)	36 (13.4)	3 (21.4)	
(+++)	149 (55.4)	5 (35.7)	
**Dog-1 (%)**			0.183
(-)	11 (4.1)	2 (14.3)	
(+)	51 (19.0)	2 (14.3)	
(++)	30 (11.2)	3 (21.4)	
(+++)	176 (65.7)	7 (50.0)	
**Ki-67 index (%)**			**< 0.001**
≤ 5 %	231 (86.2)	5 (35.7)	
> 5 %	37 (13.8)	9 (64.3)	
**PLR(%)**			0.219
≤153.31	177 (66.0)	7 (50.0)	
>153.31	91 (34.0)	7 (50.0)	
**NLR(%)**			**0.018**
≤2.81	208 (77.6)	7 (50.0)	
>2.81	60 (22.4)	7 (50.0)	
**Gastrointestinal bleeding(%)**			0.578
Yes	103 (38.4)	4 (28.6)	
None	165 (61.6)	10 (71.4)	
**Basic disease(%)**			1.000
Yes	107 (39.9)	5 (35.7)	
None	161 (60.1)	9 (64.3)	

(-), negative; (+), weakly positive; (++), moderately positive; (+++), strongly positive

### Correlation between postoperative recurrent metastasis and clinicopathological features in patients with gastrointestinal stromal tumors

To further investigate the association between recurrent metastasis and clinical features in GIST patients, univariate and multivariate Cox regression analyses were performed. The results of univariate analysis showed that postoperative recurrence or metastasis was associated with primary tumor site (*P* = 0.003, HR = 5.930, 95% CI: 1.844 ~ 19.072); tumor size (*P* = 0.015, HR = 4.852, 95% CI: 1.353~17.397); resection type (*P* = 0.041, HR = 4.759, 95% CI: 1.063~21.306); Ki67 index (*P* < 0.001, HR = 9.730, 95% CI: 2.253~29.106); NLR (*P* = 0.032, HR = 3.152, 95% CI: 1.104~8.996) and CD34 (*P* = 0.004, HR = 0.145, 95% CI: 0.039~0.541) were strongly correlated, independent of age, gender, and underlying disease (Table 2). The statistically significant differences in the above factors were included in the multivariate Cox regression model, and the primary tumor site was shown (*P* = 0.016, HR = 4.290, 95% CI: 1.307~14.082). tumor size (*P* = 0.014, HR = 4.987, 95% CI: 1.383~17.989); Ki67 index (*P*<0.001, HR = 8.198, 95% CI: 2.680~25.007) was an independent risk factor for postoperative recurrence and metastasis in low-risk GIST patients (Table 2).

**Table 2:** Univariate and multivariate Cox regression analysis in low-risk GIST patients with recurrence or metastasis

Factors	Univariate analysis		Multivariate analysis	
	HR (95%CI)	*P* value	HR (95%CI)	*P* value
**Age (%)**				
≤ 60	Reference			
> 60	0.846 (0.282~2.536)	0.766		
**Gender (%)**				
Male	Reference			
Female	0.482 (0.161~1.437)	0.190		
**Tumor site (%)**				
Stomach	Reference		Reference	
No stomach	5.930 (1.844~19.072)	**0.003**	4.290 (1.307~14.082)	**0.016**
**Resection type(%)**				
Laparoscopic resection	Reference		Reference	
Open resection	4.759 (1.063~21.306)	**0.041**	2.418 (0.420~13.926)	0.323
**Tumor size (%)**				
<4 cm	Reference		Reference	
≥4 cm	4.852 (1.353~17.397)	**0.015**	4.987 (1.383~17.989)	**0.014**
**CD117 (%)**				
(-)	Reference			
** (+)**	0.429 (0.047~3.917)	0.453		
(++)	0.244 (0.022~2.706)	0.250		
(+++)	0.307 (0.037~2.546)	0.274		
**CD34 (%)**				
(-)	Reference		Reference	
(+)	0.129 (0.024~0.704)	**0.018**	0.408 (0.071~2.346)	0.315
(++)	0.361 (0.080~1.624)	0.184	0.726 (0.144~3.655)	0.698
(+++)	0.145 (0.039~0.541)	**0.004**	0.680 (0.144~3.204)	0.626
**Dog-1(%)**				
(-)	Reference			
(+)	0.178 (0.024~1.296)	0.088		
(++)	0.680 (0.113~4.080)	0.673		
(+++)	0.288 (0.059~1.403)	0.123		
**Ki-67 index (%)**				
≤ 5 %	Reference		Reference	
> 5 %	9.730 (2.253~29.106)	**< 0.001**	8.198 (2.680~25.007)	**< 0.001**
**PLR(%)**				
≤153.31	Reference			
>153.31	1.876 (0.658~5.352)	0.239		
**NLR(%)**				
≤2.81	Reference		Reference	
>2.81	3.152 (1.104~8.996)	**0.032**	1.150 (0.344~3.849)	0.820
**Gastrointestinal bleeding(%)**				
Yes	Reference			
None	1.857 (0.582~5.924)	0.296		
**Basic disease(%)**				
Yes	Reference			
None	1.148 (0.384~3.428)	0.805		

(-), negative; (+), weakly positive; (++), moderately positive; (+++), strongly positive

### Survival curve analysis of the correlation between postoperative recurrence and metastasis and clinicopathological features in patients with gastrointestinal stromal tumor

**Fig. 2:**
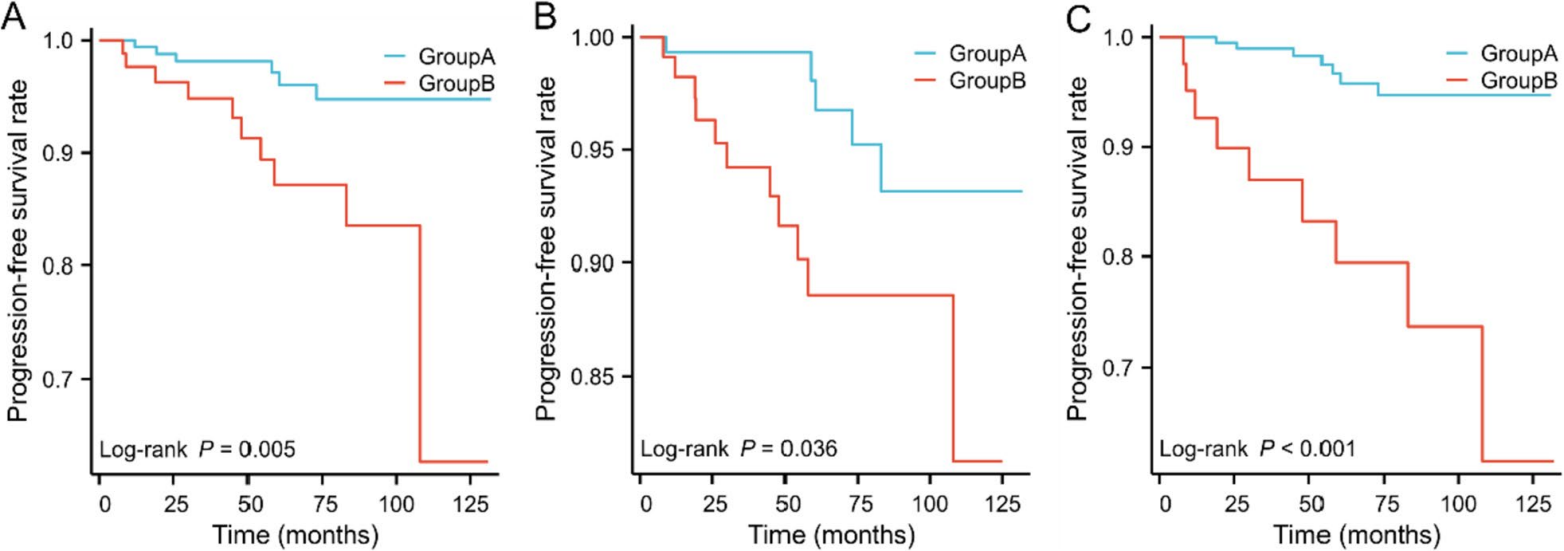
Kaplan-Meier survival curve of PFS in patients with gastrointestinal stromal tumors. **A** Primary site (Group A: stomach; Group B: no stomach). **B** Tumor size (Group A:tumor size<4cm; Group B: tumor size ≥ 4cm). **C** Ki-67 index (Group A: Ki67≤5%;Group B: Ki67>5%)

A total of 282 patients were observed during the follow-up period, which ranged from 6 to 136 months, with the last follow-up in February 2022. Further survival curve analysis showed that the primary tumor site (*P* = 0.016), tumor size (*P* = 0.014) and Ki-67 index (*P* < 0.001) had an effect on PFS in GIST patients (Fig. 2). Specifically, throughout the follow-up, the PFS rate of patients with low-risk GISTs in the primary site of the stomach was 96.86 %, which was higher than that of patients with non-gastric sites (89.01 %) (Fig. 2A) ; Furthermore, the PFS rate of low-risk GISTs with tumor size of 2-4 cm was 96.89%, and that of patients with low-risk GISTs with tumor ≥ 4 cm was 90.91% (Fig. 2B); Patients with low-risk GISTs with Ki67 ≤5% had a significantly higher PFS rate (97.03%) compared to patients with low-risk GISTs with Ki67 >5% (80.43%) (Fig. 2C). *p*-values less than 0.05 were considered statistically significant.

## Discussion

This study was a retrospective single-center investigation. The clinicopathological information of 282 patients with low-risk gastrointestinal stromal tumors who had surgical therapy at Nanjing Drum Tower Hospital was retrospectively examined. This study aimed to integrate and analyze clinicopathological data to explore the factors affecting postoperative recurrence or metastasis in patients with low-risk stromal tumors. In this study, the findings revealed that patients of different genders and age groups did not significantly differ in postoperative recurrence and metastasis, which was consistent with the results of previous studies by Nicolas Patel and Bikramjit Benipal et al. [15]. In our study, univariate analysis showed a correlation between the surgical resection modality and recurrent metastasis in patients, specifically comparing both open and laparoscopic resection modalities, and found that open resection may be associated with differences in postoperative rehabilitation and local control, which may affect the risk of recurrent metastasis. Cancer-related inflammation plays a key role in promoting tumor progression and metastasis by inhibiting the anti-tumor immune response and is closely related to various stages of tumor development [16]. Inflammatory response indicators, such as NLR and PLR, have been shown to provide useful information in predicting the prognosis of patients with multiple cancers. In recent years, many studies have supported the use of NLR as a prognostic factor for several cancer patients, including colorectal cancer, hepatocellular carcinoma, gastric cancer, and pancreatic cancer [17–19]. Goh et al. found that the clinical outcome of GIST patients was related to lymphocyte-related markers, and both NLR and PLR were proved to be independent prognostic factors for GIST [20]. Preoperative NLR is a feasible and repeatable peripheral biomarker. Incorporating it into the NIH stratification system is helpful to improve the predictive accuracy of GIST resection [21]. Our previous studies have revealed that CONUT score and serum CA125 level can be used as effective and novel indicators to predict the prognosis of patients with GIST surgery [22, 23]. This suggests that nutritional status may be an effective supplementary factor in predicting GIST recurrence when NIH risk levels are widely used. In our study, NLR cut-off values were determined by ROC curves. Univariate Cox regression showed that the higher NLR group was associated with postoperative recurrence and metastasis of low-risk stromal tumors. There was no statistical significance between PLR and postoperative recurrence and metastasis. In terms of immunohistochemistry, DOG1 and CD117 antibodies are widely considered to be the most sensitive and specific markers in the diagnosis of GIST, and CD34 is often used to distinguish GIST from other tumor biomarkers [24–26]. In our study, almost all of the GIST patients showed a positive expression of CD117, CD34 and Dog-1, and the negative rate was very low. However, only CD34 was shown to be associated with postoperative recurrent metastasis of low-risk GISTs in a univariate analysis, with positive and negative results for the other two immunohistochemical indices showing no significant differences in postoperative recurrent metastasis status.

To date, Multiple classification systems have arisen regarding patients with GIST. In 2002, Fletcher and other scholars published a consensus methodology [27]. subsequently, Miettinen presented the Armed Forces Pathology Institute (AFIP) criteria in 2006, which were derived from a long-term follow-up of 1684 cases. Tumor size and mitotic rate are taken into account in the AFIP standard, which also includes tumor site as a significant risk factor [28]. In 2008, the NIH updated the risk stratification system to include factors such as tumor site and rupture and renamed it the modified NIH criteria [8]. The newly developed standards have been widely accepted and recognized around the world. In low-risk GIST patients, an Italian cohort reported that intragastric primaries had higher recurrence risk than gastric primaries, while non-gastric primaries had slightly lower estimated DFS at 5 years [9]. Furthermore, studies by Ge et al. have shown that patients with GISTs originating from the stomach usually have a better prognosis, while patients with GISTs originating from non-stomach sites such as the small intestine and rectum, have a higher risk of recurrence and metastasis after surgery [29]. Univariate and multifactorial Cox regression analyses showed that tumor site, tumor size and Ki67 index were independent risk factors for recurrence or metastasis of GIST (*P* < 0.05). The present study further confirms that primary tumor location in a non-gastric site is an independent risk factor for recurrent metastasis after GIST, which is consistent with the results of the previous study. In the present study, the tumor size of all enrolled GIST patients was between 2-5 cm. Therefore, a new cut-off value was obtained to group the patients in this section and the optimal tumor size cut-off value was calculated to be 4.0 cm using the ROC curve. While tumor size, as a known important factor affecting the prognosis of patients with GISTs in the NIH recurrence risk grading system, similarly yielded consistent results in the univariate and multivariate analyses in this study (*P*= 0.014), suggesting that tumor size can be used as an independent risk factor for postoperative PFS in patients with low-risk GISTs. In the context of GIST, larger tumors are often accompanied by higher proliferative activity and invasiveness, and are therefore more likely to lead to disease progression and recurrence. Our study further highlights the importance of tumor size in risk stratification and treatment planning. However, given that our analysis is based on limited data from a single center, there are challenges of relatively small sample size and heterogeneity. Therefore, future studies are encouraged to consider expanding the sample size to more accurately assess the potential impact of tumor size in low-risk GIST patients.

Ki67 is a core marker of cell proliferation activity, which is closely related to the degree of malignancy, infiltration and metastasis of tumors [30, 31]. More and more evidence suggests that Ki67 may be an effective therapeutic target in cancer treatment and has been widely used as an indicator of cell proliferation in clinical practice. So far, a number of studies have reported the correlation between Ki67 expression and the malignant risk of GIST [32–34]. In this study, the median Ki67 labeling index of 5% was used as the cut-off point and patients were divided into two groups for comparison. The results showed that the recurrent metastasis rate was significantly higher in the high Ki67 labeling index group than in the low index group, while GIST patients with Ki67 >5% had significantly worse PFS during the follow-up period. These results further support the view that Ki67 index is associated with the risk of recurrent metastasis after GIST surgery. Currently, AFIP and modified NIH consensus criteria identify four key prognostic factors in GIST: location, size, mitotic index and rupture [8, 28]. Considering that the mitotic index of low-risk GIST patients was generally between 0-5/50 HPF, we did not re-grade on the mitotic term. Notably, Ki67 did not feature in the risk stratification standards for GIST. Nevertheless, our discoveries imply a crucial research significance for Ki67 with regards to recurrence, metastasis, and prognosis in patients with low-risk GISTs. Further extensive research and exploration are therefore warranted.

In summary, this study analyzed the clinical data of GIST patients, and Cox regression analysis screened out the risk factors of postoperative recurrence and metastasis in low-risk GIST patients, which provided a basis for early identification of the risk of recurrence and metastasis in patients. Kaplan-Meier survival analysis was used for survival curves of recurrent metastases after surgery for low-risk GISTs, and it was verified that tumor site, tumor size, and Ki67 index were strongly associated with patients' prognosis. These findings highlight the importance of clinicopathological features of postoperative recurrence or metastasis in patients with low-risk GISTs, and help to develop targeted interventions to improve the effective use of medical resources. Retrospective evidence suggests that the intensity of follow-up for low-risk GISTs is relatively low, and it is recommended that people with low-risk GISTs follow regular screening schedules and check carefully for signs of a second tumor at each follow-up visit [9]. Therefore, for patients with low-risk GISTs with large tumors and high Ki-67 index in non-gastric sites, it is recommended to increase the frequency of review and follow-up, and pay attention to the quality of life of patients. These findings are expected to provide more effective support for the individualized treatment of GIST patients.

Of course, we need to note that this study has some limitations. First of all, this is a single-center retrospective study, which mainly analyzed patients with low-risk GIST who underwent surgical treatment. Although we have reached some valuable conclusions, these results still need to be externally validated in a wider patient population to ensure that our conclusions are broadly applicable. Secondly, given the limited size of the sample, further confirmation through larger and multi-center clinical investigations is required to confirm the precision and dependability of our findings. Finally, our study needs to further explore other factors that may affect the prognosis of patients with GIST in order to more fully understand the complexity of this disease. Future research efforts will continue to address these issues and provide more scientific evidence for the management of GIST patients.

## Conclusion

In conclusion, the primary tumor site, tumor size, and Ki-67 index of patients with low-risk GISTs were closely associated with postoperative recurrent metastases and their prognosis. Therefore, the individualized diagnosis and treatment of low-risk GISTs should be based on a comprehensive consideration of clinicopathological features. If necessary, targeted postoperative review and follow-up management strategies should be considered to better predict and prevent postoperative recurrence and metastasis, and hopefully improve patient outcomes and quality of life.

## Data Availability

The datasets generated and analysed during the current study are not publicly available due privacy, but can be obtained by email (fengwang36@163.com) from the corresponding author on reasonable request.
